# Suprapubic and Transurethral Bladder Access for Voiding Cystourethrography in Pediatric Male Patients

**DOI:** 10.3390/pediatric16010017

**Published:** 2024-03-13

**Authors:** Wiebke Schlötelburg, Clemens Benoit, Mandy Kasper, Bernhard Petritsch, Andreas Max Weng, Thorsten Alexander Bley, Simon Veldhoen

**Affiliations:** 1 Department of Diagnostic and Interventional Radiology, University Hospital Würzburg, 97080 Würzburg, Germany; wirth_c2@ukw.de (C.B.); mandykasper@posteo.de (M.K.); petritsch_b@ukw.de (B.P.); bley_t@ukw.de (T.A.B.); simon.veldhoen@charite.de (S.V.); 2 Department of Nuclear Medicine, University Hospital Würzburg, 97080 Würzburg, Germany; 3 Institute for Diagnostic and Interventional Radiology and Neuroradiology, Bogenhausen Hospital, 81925 Munich, Germany; 4 Diagnostic and Interventional Radiology, Hospital Klagenfurt am Woerthersee, 9020 Klagenfurt, Austria; 5 Pediatric Radiology, Charité-Universitätsmedizin Berlin, corporate member of Freie Universität Berlin and Humboldt-Universität zu Berlin, 10117 Berlin, Germany

**Keywords:** voiding cystourethrography, transurethral catheterization, suprapubic puncture, radiation dose, pediatric urology, vesicoureteral reflux

## Abstract

Purpose: To compare suprapubic access (SPA) and transurethral catheterization (TUC) in voiding cystourethrogram (VCUG). Methods: Retrospective single-center evaluation of 311 VCUG performed in male patients under 12 years of age. Two study groups were built based on the bladder access method. TUC was performed in 213 patients, whereas 98 received SPA. The groups were compared regarding the procedural switch rate, the complication rate, radiation parameters, the amount of contrast media applied and the examination quality. Complications were graded in minor (contrast leakage, premature termination of the examination) and major (fever, urinary tract infection, bladder perforation). Fluoroscopy time and radiation parameters were compared. Examination quality was assessed based on the satisfactory acquisition of fluoroscopic images using a four-point Likert scale. Results: In 9% of the SPA examinations a method switch to TUC was necessary. The minor complication rate was 1.9% for TUC and 35.7% for SPA (*p* < 0.001). The major complication rate was 0.9% for TUC and 2% for SPA (*p* > 0.05). Mean fluoroscopy time and radiation dose were significantly lower in TUC (TUC, 26 ± 19 s, 0.6 ± 1.2 µGy·m^2^; SPA, 38 ± 33 s, 1.7 ± 2.9 µGy·m^2^; *p* = 0.01/0.001). There was no significant difference regarding the amount of contrast media applied (TUC, 62 ± 40 mL; SPA, 66 ± 41 mL; *p* > 0.05) and the examination quality with full diagnostic quality achieved in 88% of TUC and 89% of SPA examinations (*p* > 0.05). Conclusions: As TUC provides significantly lower radiation exposure and less periprocedural complications, it should be the primary bladder access route for VCUG in pediatric male patients.

## 1. Introduction

The genitourinary tract is the most common location of congenital malformations [1,2]. In the diagnostic workup of genitourinary malfunction, imaging is necessary for both assessing disease severity and developing adequate therapy strategies. The voiding cystourethrogram (VCUG) is a dynamic examination commonly performed in pediatric radiology enabling morphologic and functional assessment of the entire urinary tract including the renal collective systems, the ureters, the bladder and the urethra. In VCUG, fluoroscopic images are acquired at different stages of bladder filling with iodine contrast. It is the established reference technique for diagnostics of vesicoureteral reflux (VUR) in children and urethral valves in boys, respectively [3].

In children presenting with febrile urinary tract infections, 25–40% are found to have underlying VUR [4,5,6]. Persistent VUR can lead to recurrent infections and perpetual renal damage with loss of function potentially causing terminal renal failure. Posterior urethral valves (PUV) can cause hydronephrosis and are the most common cause of congenital bladder outlet obstruction in boys [7,8]. They are remnants of one or two membranes that course obliquely from the verumontanum to the lateral walls of the urethra [9] and are responsible for approximately 17% of pediatric patients with end-stage kidney disease [8,9]. In persistent reflux or when anatomic anomalies are present, surgery is necessary to eliminate VUR aiming for protection of the renal function. In this context, voiding ultrasound or VCUG are commonly used also for follow-up examinations in order to evaluate therapy success [10].

Various examination protocols for VCUG have been published. The most recent proposal published in 2016 by the American Academy of Pediatrics (AAP) aims to standardize the procedure [11]. Generally, there are two options for contrast agent application into the patient’s bladder: transurethral catheterization (TUC) and suprapubic bladder access (SPA). The AAP recommends a sterile transurethral catheterization for a standardized VCUG procedure whereas other authors favor suprapubic puncture [11,12,13,14].

To our knowledge, no study has focused on the comparison of the techniques available for bladder access in VCUG. The purpose of this research was to comprehensively compare transurethral catheterization and suprapubic bladder access.

## 2. Materials and Methods

### 2.1. Patients

In the course of our retrospective analysis encompassing a 5-year period, we scrutinized all voiding cystourethrography (VCUG) examinations administered to male subjects aged 0 to 12 within our pediatric radiology department, amounting to a total of 353 cases. The primary inclusion criterion was the availability of a comprehensive dataset encompassing radiation parameters and fluoroscopy time for each individual VCUG. Consequently, 311 VCUG examinations met the criteria, constituting a study population of 294 boys. Notably, 17 patients underwent repeat VCUG following surgical intervention for Vesicoureteral Reflux (VUR).

Transurethral catheterization (TUC) was employed in 213 cases, with a mean age of 1.45 ± 2.35 years, while suprapubic access (SPA) was utilized in 98 examinations, with a mean age of 2.46 ± 3.12 years, exhibiting no statistically significant difference (*p* > 0.05). At our institution, the preference for suprapubic puncture arises when clinical suspicion of urethral valves is strong, as evidenced by bilateral hydronephrosis, poor urine stream and bladder diverticula and when the bladder volume is sufficient for a save puncture procedure. Moreover, the decision to opt for transurethral catheterization is predominantly driven by the abovementioned criteria not being fulfilled.

Clinical indication for performing a VCUG examination was to rule out vesicourethral reflux, with 132 cases presenting with hydronephrosis (TUC/SPA: 71.2/28.8%), 115 cases involving urinary tract infection with fever (TUC/SPA: 72.2/27.8%), 12 cases with micturition disorders (TUC/SPA: 58.3/41.7%), 36 cases of renal malformation (TUC/SPA: 72.2/27.8%), 10 cases for postoperative control (TUC/SPA: 20/80%) and 6 cases for other reasons, such as post-traumatic situations (TUC/SPA: 18.7/83.3%) (Table 1). Notably, 42 cases were excluded from the study due to incomplete examination data. This comprehensive retrospective analysis provides valuable insights into the utilization patterns and clinical indications of VCUG in the pediatric male population, contributing to the refinement of diagnostic approaches in urological pediatric care.

### 2.2. VCUG Procedure

#### 2.2.1. Suprapubic Access

**Figure 1:**
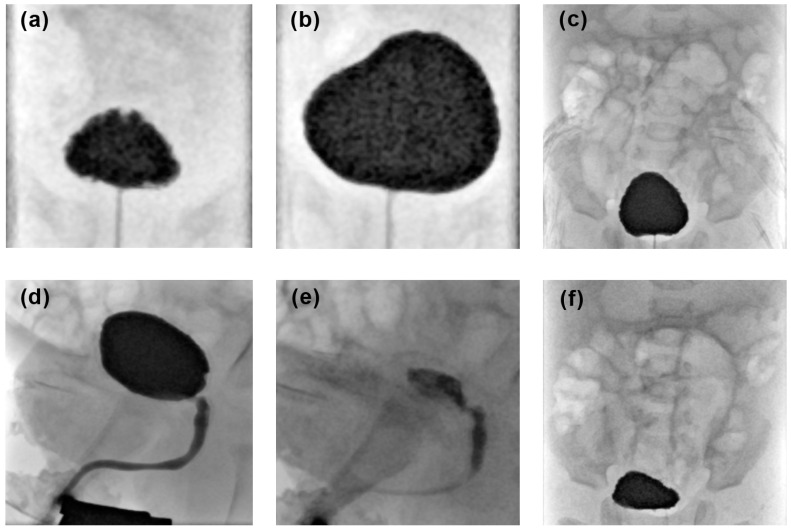
Example of a complete voiding cystourethrogram procedure: (**a**) AP bladder projections with low and (**b**) with maximum filling in supine position, (**c**) AP abdominal projection before micturition including the entire renal collective system and the bladder, (**d**,**e**) dynamic imaging during micturition including the bladder and the entire urethra in lateral view, and (**f**) AP abdominal projections after micturition including the entire renal collective system and the bladder.

1. Patient preparation:
  - Local topic anesthesia: An EMLA^®^ plaster (local anesthesia containing Lidocain and Prilocain, Aspen Germany GmbH, Munich, Germany)is applied in advance to the suprapubic region by the pediatrician or nursing staff. This helps to reduce pain and discomfort during the procedure.
2. Patient preparation:
  - The patient is positioned in the supine position on the fluoroscopy table and the plaster is removed.
  - The lower abdomen is cleaned and sterilized to minimize the risk of infection.
3. Target site identification:
  - Ultrasound-guided identification of the optimal puncture site just above the symphysis.
4. Needle insertion:
  - A sterile needle connected to an extension line (Sterican®, 20 G, 0.90 × 40 mm or 70 mm; Original Perfusor Line®, 3 × 4.1 mm × 100 cm, both: Braun®, Melsungen, Germany) is inserted through the skin and abdominal wall and directed towards the bladder. The angle and depth of insertion are guided by ultrasound imaging to ensure precision.
5. Bladder penetration:
  - The needle is advanced through the abdominal wall until it penetrates the bladder. This is confirmed by the aspiration of urine into the syringe attached to the needle. The needle is gently secured to the skin with a loosely applied plaster.
6. Sample collection:
  - Some collected sterile urine is given on a rapid urine test strip to exclude a urinary tract infection at the time of VCUG. If negative, the fluoroscopy procedure is performed. In case of a pathologic urine test strip result, the examination is terminated, the patient is referred to pediatric care for treatment of the infection and VCUG is repeated after recovery.
7. Bladder filling:
  - Following catheterization, the bladder is fractionally filled with iodine contrast (Imeron 250 or 300, Bracco Imaging Germany GmbH^®^, Konstanz, Germany) under intermittent fluoroscopy until the age-adjusted bladder capacity is reached.
  - According to the VCUG protocol proposed by the AAP Sections of Radiology and Urology the maximum bladder capacity is estimated using the following formulas [11,15]:
    $$\mathrm{for}\;\mathrm{patients}\;<\;2\;\mathrm{years}\;\mathrm{of}\;\mathrm{age}\;\mathrm{in}\;\mathrm{mL}:\;weight\left(kg\right)\times 7$$
    $$\mathrm{for}\;\mathrm{patients}\;>\;2\;\mathrm{years}\;\mathrm{of}\;\mathrm{age}\;\mathrm{in}\;\mathrm{mL}:\;age\left(years\right)\times 30+30$$
8. Image acquisition:
  - The following fluoroscopic images are acquired (Figure 1):
    1. AP bladder projections with low and maximum filling in supine position;
    2. AP abdominal projection before micturition including the entire renal collective system and the bladder;
    3. Dynamic imaging during micturition including the bladder and the entire urethra in lateral view;
    4. AP abdominal projections after micturition including the entire renal collective system and the bladder;
    5. Additional images can be obtained to document present pathological findings.
9. Post-procedure Care:
  - The needle is removed and the puncture site is dressed with a sterile bandage to prevent infection.
  - The patient may be monitored for any signs of complications, and post-procedural care instructions are provided

#### 2.2.2. Transurethral Catheterization

1. Patient preparation:
  - The patient is typically positioned on the fluoroscopy table in supine position with legs spread apart.
  - The genital area is carefully cleaned and sterilized to minimize the risk of infection.
2. Gathering equipment:
  - The healthcare provider assembles the necessary equipment, including a catheter catheter (single use catheter for children CH 06/40 or 50 cm, Uromed®, Ostssteinbek, Germany), lubricating gel, antiseptic solution and sterile gloves.
3. Gloving:
  - The doctor performing the examination uses sterile gloves to maintain aseptic conditions during the procedure.
4. Local anesthesia:
  - A local anesthetic gel is applied to the urethral opening to reduce discomfort during catheter insertion.
5. Catheter insertion:
  - The catheter is gently inserted through the urethral opening and advanced into the bladder. In boys, there is often a slight resistance during passage through the pars prostatica.
6. Urine drainage:
  - Once the catheter reaches the bladder, urine begins to drain.
  - Some collected sterile urine is given on a rapid urine test strip to rule out present infection at the time of VCUG. If negative, the fluoroscopy procedure is performed. In the case of a pathologic urine test strip result, the examination is terminated, the patient is referred to pediatric care for treatment of the infection and VCUG is repeated after recovery.
7. Catheter securement:
  - The catheter is secured in place with a with a loosely applied plaster to prevent accidental dislocation. There is usually no inflating of the small balloon at the catheter’s tip within the bladder.
8. Bladder filling and image acquisition is corresponding to points 7 and 8 of the before mentioned suprapubic access.
  - During micturition the transurethral catheter is carefully pulled out to obtain perfect overlay-free images of the urethra.

### 2.3. Examination Parameters

The following parameters were analyzed and compared for SPA and TUC:

#### 2.3.1. Procedural Switch Rate

Whenever suprapubic puncture was attempted and the examining radiologist switched to TUC during the VCUG procedure, this was documented as a periprocedural method switch and its reason was recorded as mentioned in the radiology report.

#### 2.3.2. Complication Rate

The examinations were analyzed regarding adverse events during or after VCUG. Therefore, the images, medical records and radiology reports were reviewed. Complications were graded in minor (prevesical contrast deposition due to needle dislocation and premature termination of the examination) and major (fever within two days after VCUG, urinary tract infection, bladder perforation).

#### 2.3.3. Amount of Contrast Media

The amount of contrast media applied into the bladder was taken from the radiology report.

### 2.4. Radiation Parameters

Radiation parameters were recorded from the dose report document:

- Fluoroscopy time in seconds
- Radiation dose in µGy·m^2^

### 2.5. Examination Quality

VCUG examination quality was assessed based on satisfactory acquisition of the abovementioned fluoroscopic images using a four-point Likert scale [16]:

- excellent, if all of the abovementioned images were acquired (Figure 1);
- good, if a single projection was missing;
- fair, if two projections were missing;
- non-diagnostic, if more than two abdominal projections were missing or if the lateral voiding phase—a key image series for diagnostic interpretation of VCUG—was missing.

All 311 examinations were analyzed by a radiologist with 7 years of experience. Complex cases were discussed with a senior pediatric radiologist and consensus decision was made.

### 2.6. Statistical Analyses

Statistical analyses were performed by using dedicated statistical software (SPSS Statistics v. 25, IBM, New York, NY, USA). Age, radiation dose, amount of contrast media and fluoroscopy time in the study groups are expressed as mean ± standard deviation (SD) and all data were tested for normal distribution. For data with normal distribution means were compared between the study groups using the unpaired *t*-test. Otherwise, the Mann–Whitney test was applied. For categorical variables, such as complication rates, contingency tables were built and Fisher’s exact test was used for comparison between groups. Uni- and multivariable logistic regression analyses were employed to ascertain whether the approach serves as the exclusive independent determinant for investigated parameters. All statistical tests were two sided and *p* <  0.05 was considered statistically significant.

## 3. Results

### 3.1. Examination Parameters

#### 3.1.1. Procedural Switch Rate

**Figure 2:**
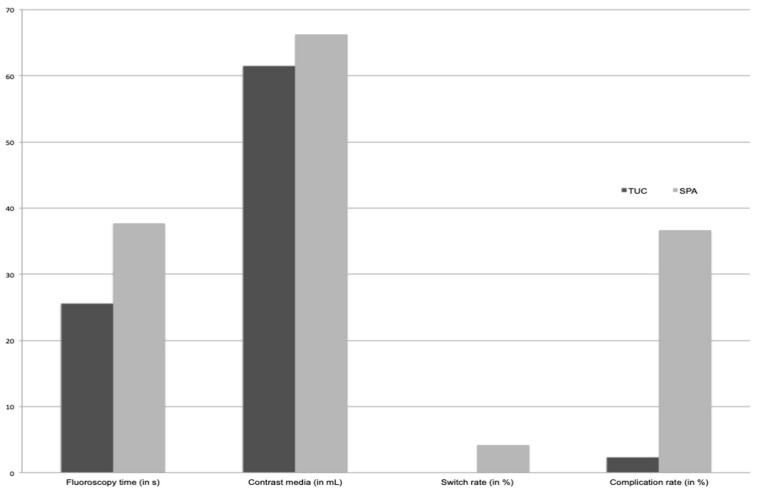
Comparison regarding transurethral catheterization (TUC) and suprapubic access (SPA) regarding fluoroscopy time (in seconds), amount of contrast media (in ml), procedural switch rate and complication rate (both in %).

A periprocedural method switch from SPA to TUC was performed in nine examinations (9%). Reasons were insufficient bladder volume for a safe puncture in seven cases (78%) and failed puncture in two cases (22%) (Figure 2 and Table 2).

#### 3.1.2. Complication Rate

Complications occurred in 1.9% of VCUG with TUC and in 35.7% with SPA, resulting in a significant difference (*p* < 0.001) (Figure 2 and Table 2).

**Figure 3:**
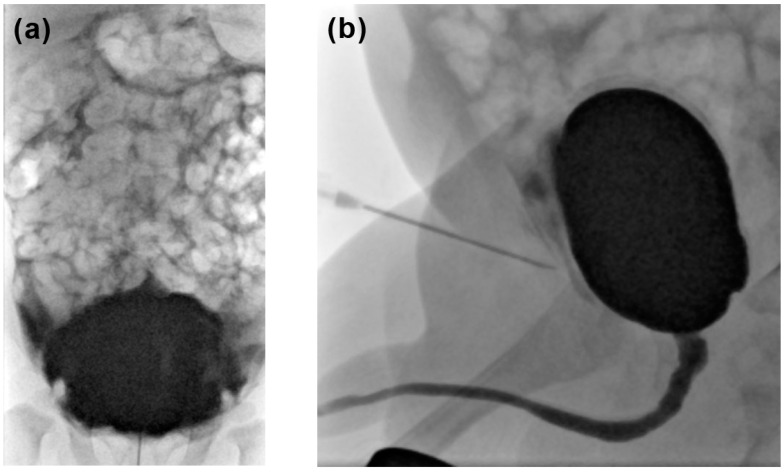
(**a**) AP abdominal projection and (**b**) lateral view: both showing paravesical contrast deposition due to needle dislocation in a patient with suprapubic access.

- Minor complications: Due to the access method, prevesical contrast leakage occurs in patients with SPA only and was found in 32 of 98 examinations performed with SPA (32.7%). Premature termination due to patient’s incompliance was necessary in two examinations performed with TUC (0.9%) and in a single examination with SPA (1.0%) (*p* > 0.05) (Figure 3).
- Major complications: There was no case of bladder perforation. Three patients had a urinary tract infection within 2 days of the VCUG procedure and were admitted to hospital: two after TUC (0.9% of all TUC-VCUG) and one after SPA. Another boy developed a fever shortly after SPA and was admitted to hospital for 3 days but no evidence for urinary tract infection was found (in total 2.0% of all SPA-VCUG) (*p* > 0.05).

#### 3.1.3. Amount of Contrast Media

The mean amount of contrast media applied was 61.5 mL ± 39.9 mL in examinations with TUC and 66.3 mL ± 41.3 mL in examinations with SPA. There was no statistically significant difference regarding the contrast amount needed between both groups (*p* > 0.05) (Figure 2 Table 2).

### 3.2. Radiation Parameters

Mean fluoroscopy time was significantly different between the groups with 25.6 s ± 19.4 s in examinations with TUC and 37.7 s ± 33.1 s in examinations with SPA (*p* < 0.01). Mean radiation dose was 0.6 ± 1.2 µGy·m^2^ in all examinations with TUC and 1.7 ± 2.9 µGy·m^2^ in all examinations with SPA, being a statistically significant difference (*p* < 0.001) (Table 2).

On univariable logistic regression analyses, the access method for VCUG is the sole predictor for the occurrence of complication and the fluoroscopy time, while on multivariable analyses, the approach remains as the only independent predictor of complication (Table 3).

### 3.3. Examination Quality

The examination quality based on the four-point Likert scale showed excellent examinations in 57% of TUC examinations (*n* = 121) and in 45% of SPA examinations (*n* = 44). Good results were achieved in 32% (*n* = 68) and in 43% (*n* = 42), respectively. Fair and non-diagnostic examination quality occurred in 3% (*n* = 6) and 8% (*n* = 18) of all examinations with TUC and in 6% (both *n* = 6) of examinations with SPA. Overall, there was no significant difference in examination quality between TUC and SPA (all *p* > 0.05).

## 4. Discussion

The purpose of this study was to compare the methods available for bladder access in voiding cystourethrography. VCUG examination protocols proposed in the literature vary regarding methodology and recommended acquisition of projections or dynamic image series [11,17]. Analyzing VCUG examinations based on the protocol used in our institution, we did not find significant differences regarding the examination quality when comparing TUC and SPA. Examinations rated good to perfect were achieved in >87% of the VCUG with both bladder access methods. VCUG has been proven to be a safe procedure in this investigation, as only 3 of 294 patients developed a postprocedural urinary tract infection and only 1 patient had fever of unknown origin. Premature termination due to incompliance was observed in three patients only. Paravesical contrast leakage was found in up to 32.7% of the SPA-VCUG being the most frequent complication in the study population. A procedural method switch from SPA to TUC was necessary in 9% of the VCUG examinations only. Reasons were insufficient bladder volume for save suprapubic puncture and failed puncture. No significant difference was found between the two access methods regarding the amount of contrast media applied. The fluoroscopy time and thus the corresponding radiation dose were significantly lower in TUC examinations.

As there is no work comparing the bladder access methods in VCUG, studies focusing on sterile urine sampling can be used for evaluation of our results as the bladder access is similar. The guidelines of the American Academy of Pediatrics and the European Society of Pediatric Urology suggest that a urine specimen be collected via SPA or TUC in febrile infants from 2 to 24 months—likewise for VCUG guidelines without favoring one of the techniques [3,18]. Pollack et al. stated that successful suprapubic bladder aspiration is primarily dependent on its volume. Therefore, the likelihood of successful SPA decreases in sick and possibly dehydrated children leading to their recommendation of primarily performing TUC [19]. The switch rate from failed suprapubic puncture to TUC was 54% in their study on 50 patients under 6 months of age being six times higher than in our study. This difference is supposed to be attributable to the fact that no ultrasound was performed for estimation of the bladder volume before puncture.

Paravesical contrast leakage was the most frequent complication in our study and can technically occur in SPA only. However, it is usually unproblematic and does not affect patient outcome. Oswald et al. found contrast leakage in 9% of all children with SPA due to needle dislocation mainly during voiding [12]. The complications defined as major are rare in VCUG and only occurred in four patients in this study (three with urinary tract infection, one with fever of unknown origin). When performed with proven sterile urine, VCUG does not cause any significant morbidity [20]. Studies focusing on voiding ultrasound have demonstrated a low periprocedural incidence of urinary tract infections. Johnson et al. found a rate of 1% in a cohort of 1203 voiding ultrasounds. Matching their results, the bladder access method was not related to UTI in the present study [21]. Iatrogenic bladder perforation being a dreaded major complication of VCUG did not occur in our study population. Costa et al. suggested that formulas based on patient age can overestimate the bladder capacity in infants and recommended weight-adjusted calculation during the first years of life as they were used in this study [22].

Several studies analyzing methods of dose reduction in VCUG have been published [17,23] and extensive work has been performed to reduce fluoroscopy dosing [24,25]. It is known that the examiner’s experience and modern fluoroscopy techniques have major impact on the radiation dose [17,26]. We attribute the higher radiation dose found in SPA to an increased number of fluoroscopy images acquired in order to assess the needle position during the filling procedure.

A recent study by Brandt et al. evaluated the experience of VCUG in 417 families in terms of children’s anxiety during the VCUG procedure, which was lower when SPA was performed instead of TUC, which contrasted with higher parental anxiety before SPA [14]. They reported better diagnostic quality using SPA, a finding we cannot confirm in our study. The study also reported a higher complication rate in patients undergoing SPA due to paravesical leakage, which is comparable to the results of the present study.

The fact that different radiologists with potentially varying individual approaches of image acquisition carried out the VCUG examinations in this retrospective analysis should be mentioned as a study limitation. A prospective study design with one radiologist performing the VCUG would have been beneficial. However, the study design chosen provides a large data set for comparison of the bladder access method in VCUG. Also, it cannot be excluded that some patients had subclinical UTI after VCUG or chose another healthcare provider for diagnostics and treatment. Therefore, the incidence of UTI might be underestimated. However, we do not expect a significant distortion of the results due to this limitation as even lower incidences of UTI after VCUG have been reported [17].

## 5. Conclusions

Compared to suprapubic bladder access, the use of transurethral catheterization for VCUG provided significantly lower fluoroscopy times and thus lower radiation exposure. Paravesical contrast leakage was found to be the most frequent minor complication of VCUG and can be avoided by choosing transurethral catheterization. As no significant differences were observed regarding other periprocedural complications, the examination quality and the amount of contrast media applied, transurethral catheterization should be the preferred bladder access method for VCUG in pediatric male patients.

## Figures and Tables

**Table 1:** Clinical indications for VCUG.

Indication	Total (*n* = 311)	TUC	SPA
Hydronephrosis	132	94 (=71.2%)	38 (=28.8%)
Urinary tract infection with fever	115	83 (=72.2%)	32 (=27.8%)
Micturition disorder	12	7 (=58.3%)	5 (=41.7%)
Renal malformation	36	26 (=72.2%)	10 (=27.8%)
Postoperative control	10	2 (=20%)	8 (=80%)
Others (e.g., post-traumatic)	6	1 (=16.7%)	5 (=83.3%)

VCUG: Voiding cystourethrogram. TUC: Transurethral catheterization. SPA: Suprapubic access.

**Table 2:** Descriptive statistics of VCUG performed with TUC and SPA.

	TUC	SPA	*p*-Value
Total no. of examinations = 311	213	98	
Age			
mean ± SD (yrs)	1.45 ± 2.35	2.46 ± 3.12	<0.01
Periprocedural Switch Rate	From SPA to TUC: *n* = 9 (9%)		
Complication rate:	4 (1.9%)	35 (35.7%)	<0.001
Major	2 (0.9%)	2 (2.0%)	>0.05
Minor	2 (0.9%)	33 (33.7%)	<0.001
Prevesical leakage	-	32 (32.6%)	
Premature termination of VCUG procedure	2 (0.9%)	1 (1.0%)	>0.05
Examination parameters:			
Radiation dose ± SD (µGy·m^2^)	0.6 ± 1.2	1.7 ± 2.9	<0.001
Contrast media amount (mL)	61.5 ± 39.9	66.3 ± 41.3	>0.05
Fluoroscopy time (s)	25.6 ± 19.4	37.7 ± 33.1	<0.01
Examination quality			
- Excellent	121 (56.8%)	44 (44.9%)	>0.05
- Good	68 (31.9%)	42 (42.8%)	>0.05
- Fair	6 (2.81%)	6 (6.1%)	>0.05
- Non-diagnostic	18 (8.4%)	6 (6.1%)	>0.05

TUC: Transurethral catheterization, SPA: Suprapubic access, SD: Standard deviation, VCUG: Voiding cystourethrogram.

**Table 3:** Uni- und multivariable logistic regression analysis.

	Univariable			Multivariable		
	OR	95% CI	*p*-Value	OR	95% CI	*p*-Value
Occurrence of complications	0.958	0.125–0.479	<0.001	1.004	1.000–1.006	0.0001
Fluoroscopy time	0.978	0.972–0.991	<0.05	0.984	0.973–0.9945	0.04
Age	0.992	0.818–0.972	0.09			
Contrast media amount	0.998	0.992–1.004	0.45			
Radiation dose	0.995	0.958–1.040	0.78			

(OR: Odds ratio, 95%CI: Confidence interval).

## Data Availability

The datasets generated during and/or analyzed during the current study are available from the corresponding author on reasonable request.
